# Altered Behavior in Encephalitis: Insights From the Australian Childhood Encephalitis Study, 2013–2018

**DOI:** 10.3389/fped.2021.667719

**Published:** 2021-12-24

**Authors:** Rebecca Burrell, Cheryl A. Jones, Philip N. Britton, The PAEDS Network, Russell C. Dale

**Affiliations:** ^1^Faculty of Medicine and Health, Sydney Medical School, The University of Sydney, Sydney, NSW, Australia; ^2^The Children's Hospital at Westmead, Sydney, NSW, Australia

**Keywords:** encephalitis, behavior and cognition, child, psychiatric, NMDA-receptor

## Abstract

Altered mental status is a major criterion for a diagnosis of encephalitis to be made with alteration in behavior, a key manifestation of altered mental status. We reviewed all evaluated cases identified by the Australian Childhood Encephalitis study between May 2013 and June 2018, to review the frequency and features of altered behavior (ALB). ALB was reported in >72% of cases of childhood encephalitis in all three major etiologic groups (infectious, immune-mediated, and unknown). The duration of ALB was >7 days in a minority, but significantly more frequent in immune-mediated compared with infectious encephalitis (27 and 10%, respectively, *p* < 0.01). ALB was most frequently characterized as irritability/agitation (47%), which predominated in children aged <1 year, and among the leading infectious causes in this age group (enterovirus, parechovirus, and bacterial meningoencephalitis). ALB in the form of disorientation/confusion (25%) was most prominent in those aged >1 year and most frequent in immune-mediated encephalitis. Hallucinations, paranoia, and aggression were all infrequent; suicidality/self-harm was not observed. ALB was reported in 20 of 21 cases of anti-*N*-methyl-d-aspartate receptor (anti-NMDAr), 19% for >7 days, and disorientation/confusion was the most frequent feature. Only one case was reported as presenting with “psychosis” and was diagnosed with anti-NMDAr encephalitis. Clinician-reported ALB is frequent but most often non-specific in childhood encephalitis. A longer duration of ALB is associated with an immune-mediated cause. More specific psychiatric symptoms (hallucinations, paranoia) are very infrequent. ALB is a hallmark of anti-NMDAr encephalitis, but psychosis is uncommon in contrast to the disorder in adults.

## Introduction

Encephalitis or inflammation of the brain comprises a heterogeneous group of neurological disorders of various etiology with protean clinical presentation. It is most often caused by infections and autoimmune processes (also known as immune-mediated encephalitis). Exceptionally, the diagnosis is made using brain biopsy; thus, several clinical or syndromic definitions have been proposed to support clinical practice and research. Widely used and endorsed definitions include those from the Brighton collaboration (1) and the International Encephalitis Consortium (IEC) (2). Altered mental status is a major criterion for the diagnosis of encephalitis in these definitions, and altered personality and/or behavior are key manifestations.

There has been considerable emphasis on subacute psychiatric presentations in patients with antibody-mediated, autoimmune encephalitis, most frequently anti–N-methyl-D-aspartate receptor (NMDAr) encephalitis (3–5). Clinical definitions for diagnosis of these immune-mediated encephalitides in adults and children highlighted the importance of altered cognition, behavioral, and psychiatric symptoms as features of autoimmune encephalitis, although with differences across the age spectrum (6, 7).

We aimed to describe the frequency and features of altered behavior (ALB) in cases of childhood encephalitis identified nationally and prospectively by the Australian Childhood Encephalitis study. We also aimed to analyze for potential associations between ALB and cause.

## Materials and Methods

Since 2013, the Pediatric Active Enhanced Disease Surveillance (PAEDS; www.paeds.org.au) network has supported the capture of prospective cases of suspected encephalitis admitted to one of seven tertiary children's hospitals for the Australian Childhood Encephalitis (ACE) study. Cases identified were evaluated by the ACE expert panel to determine if they met the criteria for encephalitis using the IEC (2) and Brighton Collaboration Encephalitis Working Group criteria (1). Where possible, the ACE expert panel also aimed to determine the underlying cause of illness and classify etiology using the recognized international standards (8).

Cases of encephalitis with viral, bacterial, fungal, or parasitic cause were classed as infectious encephalitis, whereas those with an immune-mediated cause [e.g., acute demyelinating encephalomyelitis (ADEM) and variants or anti-NMDAr encephalitis] were classed as immune-mediated encephalitis. Those with an unclear etiology were placed in a third category classed as unknown encephalitis. Cases that did not meet the IEC and Brighton criteria for encephalitis were categorized as *not encephalitis* (9).

All data from recruited ACE cases valuated by the expert panel were extracted from the ACE database, between May 2013 and June 2018 in order to undertake a descriptive analysis of ALB among cases of childhood encephalitis. The cohort was evaluated in a second stratum, with children younger than 1 year of age removed from further analysis as we considered the description of ALB in this age group potentially to be non-specific or ambiguous.

The presence or absence of ALB was collected for all recruited cases at clinical presentation, and free-text field offered data collectors the opportunity to provide detail on the nature of the ALB. Descriptive data of those cases reporting ALB were available for 71% of cases and 82% of cases over 1 year of age. Searches were performed for multiple subcategories of string data (Supplementary Table 1), accounting for potential spelling errors or alternate wording, using the filter function and creation of additional Boolean variables. Inclusion within more than one ALB string category was allowed.

Microsoft Excel (2010) was used for analysis. Median age in years was calculated along with interquartile range (IQR) for each cause and also frequency by age category. Proportion of the major etiologic groups—infectious, immune-mediated, and unknown encephalitis—were calculated with total encephalitis cases as denominator. ALB string-data category frequencies were calculated with total number of cases with available string data for each etiologic subgroup. A small number of direct comparisons of categorical data within subgroups was undertaken using χ^2^ testing to determine associations with an α value of 0.05 used to determine statistical significance.

## Results

Between May 2013 and June 2018, the ACE study identified and evaluated 777 cases of suspected encephalitis admitted at one of the PAEDS sentinel hospitals. Among these 777 cases, the expert panel concluded that 436 (56%) met the criteria for encephalitis (*n* = 341 determined not to be encephalitis). Ages of the cases ranged from newborn (day 0 of life) to 15.02 years (Table 1). Those with infectious encephalitis tended to be younger in age (1.5 years, IQR = 7.05 years) compared with those with immune-mediated (7.3 years, IQR = 7.93 years) or unknown encephalitis (6.4 years, IQR = 7.84 years). Cases that did not meet the criteria for encephalitis (i.e., not encephalitis group) also tended to be younger in age (1.3 years, IQR = 5.45 years) (Table 1). In total, 285 cases were aged <1 year.

**Table 1:** Age and sex distribution by etiologic group of children with suspected encephalitis identified by the Australian Childhood Encephalitis study, 2013–2018.

	**Encephalitis**				
	**Infectious**	**Immune**	**Unknown**	**Total encephalitis**	**Not encephalitis**
*N*	256 (59)	97 (22)	83 (19)	436	341
Sex, male, *n* (%)	147 (57)	53 (55)	45 (54)	245 (56)	197 (58)
Age, Median Inter quartile range (IQR) (years)	1.5 (7.05)	7.3 (7.93)	6.4 (7.84)	2.6 (7.98)	1.3 (5.45)
<1, *n* (%)	110 (43)	4 (4)	11 (13)	125 (29)	160 (47)
1–4, *n* (%)	67 (26)	24 (25)	28 (34)	119 (27)	85 (25)
5–9, *n* (%)	42 (16)	34 (35)	22 (27)	98 (22)	49 (14)
10–14, *n* (%)	37 (14)	35 (36)	46 (55)	118 (27)	46 (13)
15–19, *n* (%)	0 (0)	0 (0)	0 (0)	0 (0)	1 (0.3)

Of the 436 cases with encephalitis, a majority (59%) had an infectious cause, and 22% had an immune-mediated cause, leaving 19% of encephalitis cases with an unknown cause (Table 1). Among those with immune-mediated encephalitis, two causes predominated with 52 cases (69%) determined to be ADEM and 20 (27%) to be anti-NMDAr encephalitis.

ALB was reported to be a feature of encephalitis presentation at high frequency (>70%) in all etiologic subgroups, and this remained similarly frequent when children younger than 1 year were removed (Table 2). The frequencies of those cases with prolonged ALB (>7 days at presentation) were more varied among the subgroups, with highest frequency (27% aged >1 year) among cases of immune-mediated encephalitis (Table 2), and significantly more frequently than in cases of infectious encephalitis (10%, *p* < 0.01).

**Table 2:** Altered level of behavior (ALB) among children with suspected encephalitis identified by the Australian Childhood Encephalitis study, 2013–2018.

		**Encephalitis**, ***n*** **(%)**				
		**Infectious**	**Immune**	**Unknown**	**Total encephalitis**	**Not encephalitis**
All cases
	ALB	179 (72)	75 (78)	57 (72)	311 (74)	255 (79)
	ALB > 7 days	11 (6)	19 (25)	7 (12)	37 (12)	33 (13)
	Total with descriptive data	172	68	55	295	244
	ALB + irritability/agitation	86 (50)	28 (41)	26 (47)	140 (47)	159 (65)
	*ALB* + *disorientation/confusion*	31 (18)	27 (40)	16 (29)	74 (25)	34 (14)
	*ALB* + altered speech^*^	12 (7)	13 (19)	7 (13)	32 (11)	14 (6)
	ALB + hallucinations	4 (2)	3 (4)	2 (4)	9 (3)	6 (2)
	ALB + paranoia	0 (0)	1 (1)	0 (0)	1 (0)	1 (0)
	ALB + suicidality/self-harm	0 (0)	0 (0)	0 (0)	0 (0)	0 (0)
	*ALB* + *aggressive/combative*	3 (2)	5 (7)	7 (13)	15 (5)	18 (7)
	ALB + sleep disturbance	2 (1)	6 (9)	3 (5)	11 (4)	6 (2)
	ALB + fever	142 (81)	43 (60)	40 (73)	225 (76)	184 (73)
Age ≥1 y
	ALB	100 (71)	71 (77)	51 (73)	222 (74)	124 (76)
	ALB >7 days	10 (10)	19 (27)	7 (14)	36 (16)	31 (25)
	Total with descriptive data	95	64	49	208	119
	ALB + irritability/agitation	39 (41)	25 (39)	21 (43)	85 (41)	53 (45)
	*ALB* + *disorientation/confusion*	31 (33)	27 (42)	16 (33)	74 (36)	34 (29)
	*ALB* + altered speech^*^	12 (13)	13 (20)	7 (14)	32 (15)	14 (12)
	ALB + hallucinations	4 (4)	3 (5)	2 (4)	9 (4)	6 (5)
	ALB + paranoia	0 (0)	1 (2)	0 (0)	1 (0)	1 (1)
	ALB + suicidality/self-harm	0 (0)	0 (0)	0 (0)	0 (0)	0 (0)
	*ALB* + *aggressive/combative*	3 (3)	5 (8)	7 (14)	15 (7)	17 (14)
	ALB + sleep disturbance	2 (2)	6 (9)	3 (6)	11 (5)	6 (5)
	ALB + fever	79 (82)	39 (57)	38 (78)	156 (75)	76 (62)

* *The authors acknowledge that altered speech as reported here is not specific and could result from a multiplicity of processes including a range of pathologies affecting articulation (dysarthria) and/or language (dysphasia)*.

Where a specific description of the nature of ALB was reported (71% of all cases and 82% aged >1 year), irritability/agitation was the most common descriptor and reported with similar frequency in all etiologic categories, and the frequency was reduced across categories when children younger than 1 year were removed from the analysis (Table 2).

The next most common feature of ALB, and most frequent in immune-mediated encephalitis (42% aged >1 year; Table 2), was disorientation/confusion albeit not significantly more frequent than in infectious encephalitis (33% aged >1 year). Altered speech occurred in a minority of cases but was most frequent in immune-mediated encephalitis (20% all; 23% anti-NMDAr encephalitis), although again not significantly more frequent than in infectious encephalitis (13%; Table 2).

Hallucinations were reported infrequently and with similar frequency across the groups, and children who experienced hallucinations were most often also reported to be disoriented and confused (12/18). Among cases where hallucinations were reported, infectious causes included influenza (*n* = 2), post-transplant human herpesvirus type 6 limbic encephalitis (*n* = 1), and Epstein–Barr virus (*n* = 1); immune-mediated causes included ADEM (*n* = 2); and anti–voltage-gated potassium channel encephalitis (*n* = 1). There were a further two cases of encephalitis with unknown cause.

Paranoia was less frequent, with only two cases reported. These two children were aged 14.8 and 7.7 years with expert panel diagnoses of anti-NMDAr encephalitis and “not encephalitis—behavioral disturbance not specified,” respectively. Both were aggressive, and the child with anti-NMDAr encephalitis also had sleep disturbance and irritability and was disoriented. A further child was described as having a “psychotic episode,” including being physically aggressive. This child was 13.9 years old and also determined to have anti-NMDAr encephalitis. There were no cases reported in which self-harm or suicidality was reported as a feature of ALB.

ALB with concurrent fever at presentation was common in all groups (>50%), but considerably less frequent in the immune-mediated encephalitis group than in the infectious encephalitis group (57 vs. 82%, respectively; *p* < 0.01).

**Figure 1:**
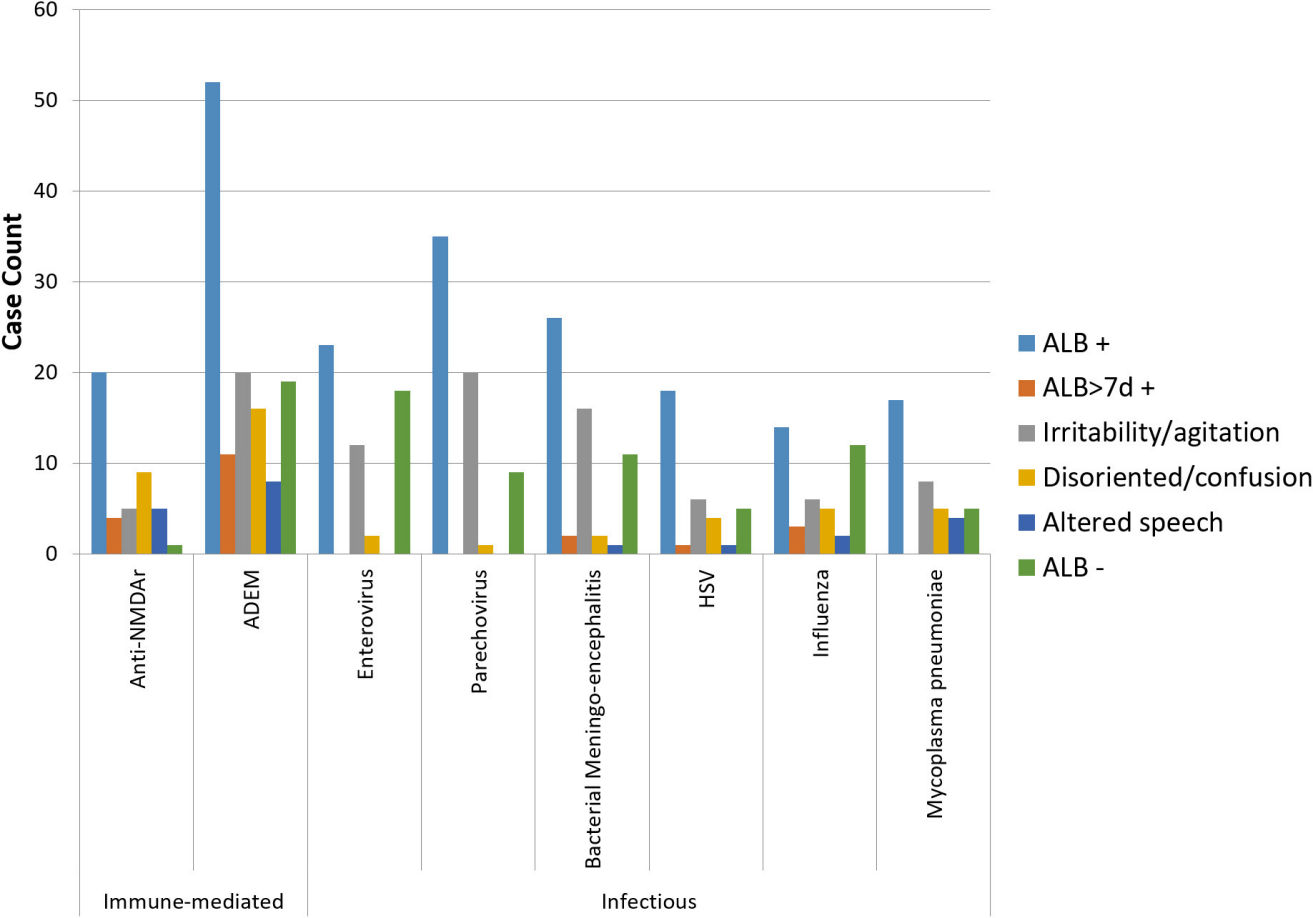
Bar graph showing the frequency of ALB with its main descriptors among the leading infectious and immune-mediated causes of encephalitis (*n* > 20) identified by the Australian Childhood Encephalitis study, 2013–2018. HSV, herpes simplex virus.

Figure 1 shows the frequency of ALB and its main descriptors among the leading causes of childhood encephalitis identified by the ACE study. ALB was most frequent among cases on anti-NMDAr encephalitis (95%; 20/21 cases). It was reported in 53–79% of other causes. Irritability/agitation predominated among cases of enterovirus, parechovirus, and bacterial meningoencephalitis, all leading causes in young infants. Disorientation/confusion was only reported more frequently than irritability/agitation among cases of anti-NMDAr encephalitis. ALB of more than 7 days occurred in 19 and 15% of cases of anti-NMDAr and ADEM, respectively, and occurred in between 0 and 11% among the infectious causes. Median days of ALB was 3 and 4 days for anti-NMDAr and ADEM, respectively.

## Discussion

We have reported the spectrum of ALB reported among cases of childhood encephalitis from a nationally ascertained and comprehensively evaluated Australian cohort. Several key findings emerge. Clinician-reported ALB—a hallmark of possible encephalitis—was frequent but most often non-specific, especially among young children where irritability/agitation was prominent. More specific psychiatric symptoms (hallucinations, paranoia) were very infrequent. Where ALB is present and encephalitis diagnosed, a prolonged duration (>7 days) and a relative absence of fever appear to correlate with an increased likelihood of an immune-mediated cause of the encephalitis.

Cellucci et al. recently published a modification of clinical diagnostic criteria for adult autoimmune encephalitis to be applied to children (7). Our data are consistent with their recommendations in showing very high frequency of ALB in the form of disorientation/confusion and/or irritability in childhood anti-NMDAr, but relative infrequency of frank psychosis. These findings build on comparable data from two recent systematic reviews (5, 10). Notably, despite an emphasis on the subacute nature of anti-NMDAr encephalitis in adults (6), our data suggest that ALB is still relatively acute in affected children (median duration of 3 days).

Although infrequent, hallucinations were evenly distributed across infectious and immune-mediated causes of encephalitis. This points to a need for nuance with respect to a focus on psychiatric features as manifestations of autoimmune encephalitis.

Our dataset did not allow us to quantify the frequency of encephalitis among children who present with ALB acutely to children's hospitals. The low incidence of encephalitis at a population level (and our anecdotal experience) would suggest that encephalitis likely contributes to only a very small proportion. Further, we acknowledge that the ACE study was not designed to systematically collect detailed or longitudinal clinical data during illness episodes; however, the data collection form offered opportunity for additional comments to be recorded based on clinician assessment at presentation, and this opportunity was taken up in a majority, but not all cases, with respect to the nature of ALB. We have assumed that where a feature was not reported in these comments, it was not present. Notwithstanding these limitations, the descriptive analysis presented provides a cross-sectional picture of the features of ALB among childhood encephalitis cases of all causes that can inform future work in this area.

## Data Availability Statement

The datasets presented in this article are not readily available because the ACE study dataset remains under the governance of the ACE study investigators and is the subject of ongoing analyses. Requests to access the datasets should be directed to philip.britton@health.nsw.gov.au.

## Ethics Statement

The studies involving human participants were reviewed and approved by Sydney Children's Hospitals Network - Human Research Ethics Committee (2019/ETH06144). Written informed consent from the participants' legal guardian/next of kin was not required to participate in this study in accordance with the national legislation and the institutional requirements.

## The PAEDS Network Investigators

Russell C. Dale, Christopher C. Blyth, Julia E. Clark, Nigel Crawford, Helen Marshall, Elizabeth J. Elliott, Kristine Macartney, Robert Booy, Nicholas Wood, Peter McIntyre, Jim Buttery, Anne Kynaston, Peter Richmond, and Joshua Francis.

## Author Contributions

CJ conceptualized the Australian Childhood Encephalitis (ACE) study. PB and CJ conceptualized this analysis. RB and PB performed the analysis. RB wrote the initial draft. All authors reviewed and revised the manuscript.

## Funding

This work was supported by the Australian Commonwealth Department of Health and the National Health and Medical Research Council (NHMRC) including through the NHMRC Centre for Research Excellence in Critical Infections [Grant (GNT) 1001021] to CJ, and the NHMRC Centre for Research Excellence in Emerging Infectious Diseases to CJ (GNT1102692). PB was supported by an NHMRC Postgraduate Fellowship (GNT1074547) and Early Career Fellowship (GNT1145817).

## Conflict of Interest

The authors declare that the research was conducted in the absence of any commercial or financial relationships that could be construed as a potential conflict of interest.

## Publisher's Note

All claims expressed in this article are solely those of the authors and do not necessarily represent those of their affiliated organizations, or those of the publisher, the editors and the reviewers. Any product that may be evaluated in this article, or claim that may be made by its manufacturer, is not guaranteed or endorsed by the publisher.
