# Supplementary material for: Altered Behavior in Encephalitis: Insights From the Australian Childhood Encephalitis Study, 2013–2018
Source: Front Pediatr. 2021 Dec 24;9:667719. doi: 10.3389/fped.2021.667719 (PMC8739917; doi:10.3389/fped.2021.667719)
Supplement: Supplementary file 1 [file Table_1.docx]

Table S1 shows search terms (with truncation) used to identify and categorise description of altered behaviour in free-text (string data) fields in the ACE dataset.

**Supplementary Table 1.**

| **Category** | **String Search Terms** |
| --- | --- |
| Irritability/Agitation | irrit*, agit*, irit*, aggit* |
| Disorientation/Confusion | disor*, confu* |
| Altered Speech | spee*, spea*, talk* |
| Hallucinations | hallu*, halu* |
| Paranoia | para*, parra* |
| Suicidality/Self Harm | suici*, harm* |
| Aggressive/Combative | aggre*, combat*, agre* |
| Sleep Disturbance | sleep* |
